# Novel tRNA Gene Rearrangements in the Mitochondrial Genomes of Poneroid Ants and Phylogenetic Implication of Paraponerinae (Hymenoptera: Formicidae)

**DOI:** 10.3390/life13102068

**Published:** 2023-10-16

**Authors:** Zijun Xiong, Ding He, Xuanming Guang, Qiye Li

**Affiliations:** 1 College of Life Sciences, University of Chinese Academy of Sciences, Beijing 100049, China; xiongzijun@genomics.cn; 2 BGI Research, Wuhan 430074, China; 3 Section for Ecology and Evolution, Department of Biology, University of Copenhagen, 2100 Copenhagen, Denmark; dinghe@biosustain.dtu.dk; 4 BGI Research, Shenzhen 518083, China; 11707016@zju.edu.cn

**Keywords:** *Paraponera clavata*, *Harpegnathos venator*, *Buniapone amblyops*, mitochondrial genome, gene rearrangement, phylogeny

## Abstract

Ants (Formicidae) are the most diverse eusocial insects in Hymenoptera, distributed across 17 extant subfamilies grouped into 3 major clades, the Formicoid, Leptanilloid, and Poneroid. While the mitogenomes of Formicoid ants have been well studied, there is a lack of published data on the mitogenomes of Poneroid ants, which requires further characterization. In this study, we first present three complete mitogenomes of Poneroid ants: *Paraponera clavata*, the only extant species from the subfamily Paraponerinae, and two species (*Harpegnathos venator* and *Buniapone amblyops*) from the Ponerinae subfamily. Notable novel gene rearrangements were observed in the new mitogenomes, located in the gene blocks *CR-trnM-trnI-trnQ-ND2*, *COX1-trnK-trnD-ATP8*, and *ND3-trnA-trnR-trnN-trnS1-trnE-trnF-ND5*. We reported the duplication of tRNA genes for the first time in Formicidae. An extra *trnQ* gene was identified in *H. venator*. These gene rearrangements could be explained by the tandem duplication/random loss (TDRL) model and the slipped-strand mispairing model. Additionally, one large duplicated region containing tandem repeats was identified in the control region of *P. clavata*. Phylogenetic analyses based on protein-coding genes and rRNA genes via maximum likelihood and Bayes methods supported the monophyly of the Poneroid clade and the sister group relationship between the subfamilies Paraponerinae and Amblyoponinae. However, caution is advised in interpreting the positions of Paraponerinae due to the potential artifact of long-branch attraction.

## 1. Introduction

Ants (Hymenoptera: Formicidae) are among the most abundant insects inhabiting most of the terrestrial surface of the earth, consisting of 17 extant subfamilies and over 17,000 species (https://www.antweb.org/ (accessed on 1 August 2023)) [1]. Ants are well known among insects for their ecological dominance as generalist and specialist predators, scavengers, omnivores, granivores, and indirect herbivores, and are the most ecologically dominant of all eusocial insects [2]. The relationships among Formicidae promote a deep understanding of why ants have become so successful. The phylogenetic relationship of the Formicidae subfamily has been investigated in a series of studies [3,4,5,6,7,8,9,10,11]. Formicidae was subdivided into three major clades: the Formicoid, Leptanilloid and Poneroid. The Poneroid clade consists of six ant subfamilies, namely Agroecomyrmecinae, Amblyoponinae, Apomyrminae, Paraponerinae, Ponerinae, and Proceratiinae [12]. While the monophyly of the Poneroid clade is widely accepted [5,6], the phylogenetic relationships among the Poneroid subfamilies, particularly Paraponerinae, remain uncertain [4,5,6]. *Paraponera clavata*, is the only extant species of the subfamily Paraponerinae. It is one of the most dangerous animals to human beings in the tropical rainforests of Central and South America [13]. It is commonly known as the bullet ant due to its extremely painful sting. The sting is the most painful of all Hymenoptera [14], which is said to be comparable to being shot with a bullet. 

Mitochondrial genomes (mtDNA) are widely used in species identification, population genetics, and evolutionary and phylogenetic studies [15,16,17,18,19]. The mtDNA of insects is usually a typical circular molecule with a length of 14~20 kb and contains 13 protein-coding genes (*cytb* for cytochrome b, *cox1-cox3* for cytochrome oxidase subunits 1–3, *atp6* and *atp8* for ATP synthase subunits 6 and 8, respectively, and *nad1-nad6* and *nad4L* for NADH dehydrogenase subunits 1–6 and 4 L, respectively), 22 transfer RNA genes (1 for each amino acid, except for leucine and serine, which have 2 genes), and 2 ribosomal RNA genes (*rrnL* and *rrnS* for large and small rRNA subunits), and 1 major noncoding region [15]. The development of genome skimming techniques has revolutionized the cost-effective next-generation sequencing of mitochondrial genomes, even from samples with high-density genomic DNA [20]. Additionally, mitochondrial DNA (mtDNA) can be obtained as a by-product during whole genome sequencing. Consequently, there has been a substantial increase in the number of released insect mitochondrial genomes in recent years [21]. Mitochondrial genomes have played a significant role in elucidating the phylogenetic relationships within insects [16,22,23]. However, there is a notable scarcity of complete mitochondrial genomes for ants, with only 86 species from 9 ant subfamilies having complete mitochondrial genome sequences deposited in GenBank (accessed on 13 July 2023), complete mitogenomes of some ant subfamilies, such as Paraponerinae, are still missing. This inadequacy is particularly noticeable in certain ant subfamilies such as Ponerinae, which is the third largest subfamily with 47 genera and more than 1200 species, where only 4 complete mitogenomes have been published [24]. This limited number is considerably lower compared to other insect groups with similar species diversity.

The majority of insect mitogenomes exhibit a typical gene order. However, as the decoding of insect mitogenomes has advanced, researchers have discovered novel gene rearrangements in various insect groups, including Hymenoptera [25], Hemiptera [26,27], Mantodea [28,29], Coleoptera [30], and Diptera [31]. These gene rearrangements often involve duplication, translocation, inversion, and pseudogenization of tRNA genes. The tandem duplication/random loss (TDRL) model [32] and the slipped-strand mispairing model [33] have been widely employed to explain the mechanisms behind these gene rearrangements. According to this model, the duplication of a tandem segment of genes is the result of slipped-strand mispairing or inaccurate termination during replication. Subsequently, random deletion of some duplicated genes leads to the creation of a novel gene order.

In this study, we took advantage of whole genome sequencing linked-reads and first presented three complete mitogenomes of Poneroid ants: the only extant species from the subfamily Paraponerinae *(Paraponera clavate)*, and two species from the Ponerinae subfamily (Jerdon’s jumping ant, *Harpegnathos venator*, and the only species from the genus *Buniapone, Buniapone amblyops*). We were particularly focused on (1) characterizing the new mitogenomes of species belonging to the ‘Poneroid’ clade; (2) proposing a hypothetical process for gene rearrangements using the tandem duplication/random loss (TDRL) model and the slipped-strand mispairing model; and (3) discussing the phylogenetic relationships among Formicidae by incorporating the newly sequenced mitogenomes. Our study aims to contribute to our understanding of gene rearrangements and the phylogenetic relationships within the Formicidae family.

## 2. Materials and Methods

### 2.1. Sample Collection and DNA Extraction

Specimens of *P. clavata* workers were collected in the rainforest of French Guiana, South America (−59.7049 longitude, 5.10337 latitude). Samples were preserved in 75% ethanol and sent to the University of Copenhagen for DNA extraction. Specimens of *H. venator* were collected from Lingshan County, Qinzhou City, Guangxi Zhuang Autonomous Region, China (109.11 longitude, 22.33 latitude). Specimens of *B. amblyops* were collected from Nanan County, Quanzhou City, Fujian Province, China (118.486499 longitude, 25.202839 latitude). The colonies were brought back to the laboratory of the Kunming Institute of Zoology, Chinese Academy of Sciences, China. Genomic DNA was extracted using an improved phenol/chloroform phase separation protocol [34]. DNA quality was measured using agarose gel electrophoresis and pulse electrophoresis. DNA concentration was determined using Qubit. Qualified DNA was used to build a stLFR (single-tube long fragment reads) library and sequenced on the DNBSEQ platform (BGI, Shenzhen, China) [35]. 

### 2.2. Mitogenome Assembly and Annotation

We obtained a total of 132 Gb, 64 Gb, and 73 Gb of short reads for *P. clavata*, *H. venator*, and *B. amblyops*, respectively. These reads had an insert size of 250 bp and a read length of 100 bp. Low-quality reads (reads with adapter sequences and anonymous bases) were filtered with SOAPnuke [36]. The GetOrganelle pipeline [37] was used to recruit mitochondrial genome reads from clean data. Clean reads were then assembled and circularized to obtain the complete mitochondrial chromosome of *P. clavata*, *H. venator*, and *B. amblyops* with a coverage of >200×. Clean reads were mapped back up to the assembly using Geneious v.10.1.3 (http://www.geneious.com/ (accessed on 1 December 2021)) to check accuracy. The genes of mitogenomes were annotated using MitoZ [38] and MITOS [39]. The circular mitogenome map was drawn using the online web tool CGView [40]. The secondary structure of tRNAs was generated in the MITOS web server. 

### 2.3. Sequence Analysis

Nucleotide composition and relative synonymous codon usage (RSCU) were calculated using MEGA [41]. The bias of AT and CG were calculated according to the formulas AT skew = (A − T)/(A + T) and GC skew = (G − C)/(G + C). To visualize the gene order of all 37 mitochondrial genes (protein-coding genes, PCGs; transfer RNAs, tRNAs; and ribosomal RNAs, rRNAs) across ant subfamilies, we ordered genes starting with the *Cox1* gene in a linear view. We used AliGROOVE [42] to test the heterogeneous sequence divergence within and among different ant groups. Indels in the PCG sequence alignment dataset were treated as ambiguities. A BLOSUM62 matrix was used for scoring and pairwise sequence distances were generated. The distances were then compared to overall distances across the data matrix. 

### 2.4. Phylogenetic Analysis

We obtained complete mitogenomes of ants from the GenBank database. We chose 1 representative record for each species, resulting in a total of 89 mitochondrial genomes representing 9 ant subfamilies. Two bee species were selected as outgroups for comparison. Nucleotide and amino acid (AA) sequences of the 13 PCGs, nucleotide sequences of PCGs, and 2 rRNA genes (PCGRNA) from mitogenomes were aligned independently using Mafft [43] with default parameters and then concatenated to form a super alignment using a customized Perl script. Maximum likelihood (ML) inference using IQ-Tree 2 [44] was used for model-based inference of phylogeny. The models GTR + F + R8 and mtInv + F + R9 were determined as the best models for PCG/PCGRNA and AA datasets using the program Modelfinder [45] according to the Bayesian information criterion (BIC), respectively. Maximum likelihood phylogenetic analysis was employed using IQ-Tree 2 under the best model with 1000 bootstrap replicates for each dataset. To reduce the effect of sequence heterogeneity, Bayes inference (BI) was conducted using PhyloBayes MPI [46] with a site-heterogeneous CAT + GTR model. Two independent chains were performed until the likelihoods stabilized and the two chains converged with a maximum discrepancy between bipartitions < 0.3. Initial trees of each run were discarded as burn-in, and the remaining trees were used to generate a consensus tree.

## 3. Results and Discussion

### 3.1. General Characteristics of Three Mitochondrial Genomes

**Figure 1:**
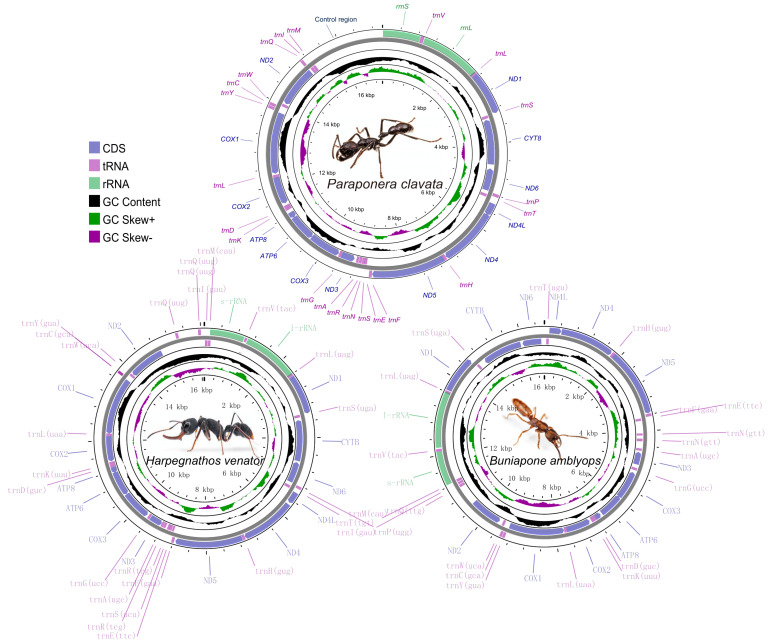
Graphical map of the complete mitochondrial genome of *Paraponera clavata, Harpegnathos venator, and Buniapone amblyops*. The length scale and the photo of species are indicated by the innermost circle. Arrows indicate the gene transcription direction, with clockwise representing the heavy strand and counterclockwise representing the light strand. GC skew is plotted using a green and purple sliding window, indicating positive and negative values, respectively. GC content shows deviation from the average GC content of the entire sequence. The typical genes are shown in standard abbreviations.

The sizes of the mitochondrial genomes for *P. clavata*, *H. venator*, and *B. amblyops* were found to be 17,018 bp, 16,089 bp, and 16,621 bp, respectively. These sizes fall within the range of previously published ant mitogenomes (15,310–19,464 bp, Table S1). The mitochondrial genome of *P. clavata* exhibited the typical structure found in ants, consisting of 13 protein-coding genes (PCGs), 2 rRNA genes, 22 tRNA genes, and a large control region (Figure 1). In the case of *B. amblyops*, a loss of the *trnP* gene was observed from its original location in the mitogenome, which is typically found between *ND6* and *trnT*. This resulted in an intergenic region of 129 bp. Upon further analysis, a 68 bp segment within this region displayed high sequence similarity (~93% sequence identity) to *trnP* genes from other mitochondrial genomes (Figure S1). The *trnP* sequence in *B. amblyops* was found at the edges of the assembled mitochondrial sequences, making it challenging to identify using MitoZ [38] and MITOS [39]. However, after correction, it was determined that *B. amblyops* does possess the typical 37 genes and a control region. 

In the mitochondrial genome of *H. venator*, an additional *trnQ* gene (named trnQ2) was observed alongside the typical set of 22 tRNA genes. Both *trnQ1* and *trnQ2* were encoded by the heavy strand and were situated between *Nad2* and the *rRNA* genes (Figure 1). Notably, the sequences of *trnQ1* and *trnQ2* were identical, with a length of 69 bp. These two tRNA genes are considered paralogs, and one copy was likely created through gene duplication.

Among the three mitogenomes, four protein-coding genes (*Nad1*, *Nad4*, *Nad4l*, and *Nad5*) were located on the heavy strand, while the remaining nine genes (*Cox1*, *Cox2*, *Atp8*, *Atp6*, *Cox3*, *Nad3*, *Nad6*, *Cytb*, and *Nad2*) were encoded on the light strand (Figure 1). All protein-coding genes initiated with the standard ATN codons for translation initiation and terminated with the TAA/TAG stop codon. However, there were a few exceptions: the *ATP6* gene in *P. clavata* ended with TA, *Nad2* and *Nad5* in *H. venator* ended with T, and *Nad2* and *Cox1* in *B. amblyops* ended with T and TA, respectively (Tables S2–S4). The presence of incomplete stop codons can be explained by the punctuation model for mature mRNA processing, followed by 3’ polyadenylation [47].

Gene overlaps were identified at specific gene boundaries (Tables S2–S4). In *H. venator* and *P. clavata*, an overlap between the *Atp6* and *Atp8* genes was observed, while in *B. amblyops*, this overlap was not present. Similarly, an overlap between the *Nad4l* and *Nad4* genes was found only in *P. clavata*. These overlapping gene structures have also been documented in other Formicidae subfamilies, such as Dolichoderinae [48]. The gene junctions of *Atp8/Atp6* and *Nad4l/Nad4* are considered conserved features in insect mitogenomes [47,49]. The conservation of these overlaps is hypothesized to be associated with the bicistronic expression of these gene clusters. The transcription of mitochondrial protein-coding genes in insects generates 11 mature RNA transcripts. Among them, two transcripts are polycistronic, representing the gene clusters *Atp8/Atp6* and *Nad4l/Nad4*, while the remaining transcripts are monocistronic [47,49].

**Figure 2:**
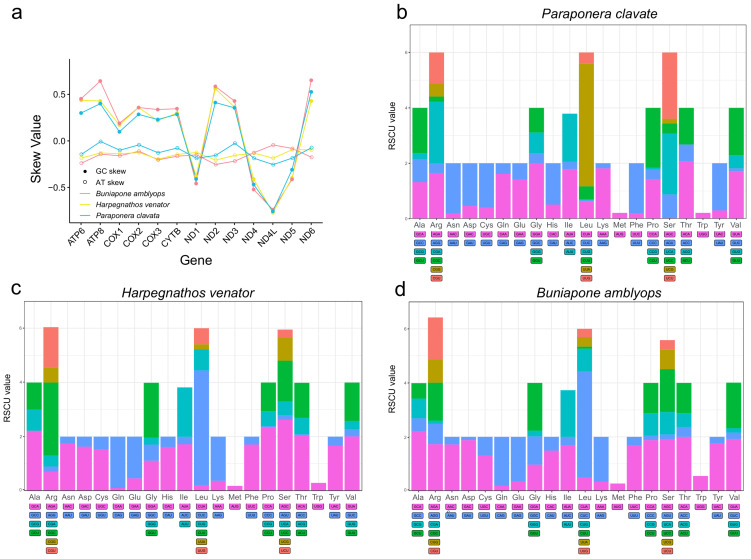
(**a**) Distribution of AT skew and GC skew in PCGs of three mitochondrial genomes. (**b**–**d**) Relative synonymous codon usage (RSCU) of PCGs of three mitogenomes. The degenerate synonymous codons are shown on the *x*-axis, and the RSCU values are shown on the *y*-axis.

The nucleotide composition of the three new mitogenomes revealed an AT-biased pattern, with AT percentages ranging from 80.1% to 82.1% (Table 1). This AT bias is a common characteristic observed in insect mitogenomes [16,50]. The AT skew was found to be positive in two Ponerinae mitogenomes and slightly negative in *Paraponera clavata*, while an obvious negative GC skew (−0.32, −0.34, and −0.43) was observed across all three mitogenomes, suggesting a preference for the C to G base usage (Table 1). The AT skews of the protein-coding genes were highly consistent among the three ant mitogenomes. The protein-coding genes on the light strands exhibited positive GC skews, while those on the heavy strands (*ND1*, *ND4L*, *ND4*, and *ND5*) showed negative GC skews (Figure 2).

The base bias observed in the mitogenomes was also evident in the codon usage. AT-rich codons were found to be the most frequently used. The first five most abundant amino acids (TTT, TTA, ATT, TAT, and ATA) in the mitogenomes were encoded by A + T-rich codons. This AT-biased pattern was also present in the terminal codons, where 31 out of the 39 protein-coding genes had the TAA codon as the terminal codon (Tables S2–S4). The codon bias was further demonstrated through the relative synonymous codon usage (RSCU) values. These values provide insight into the non-randomness of codon usage by comparing the frequency of a specific codon with the frequency of synonymous codons for the same amino acid. The common pattern observed in the amino acids corresponded to NNA or NNU codons, with RSCU values predominantly > 1 (Figure 2). Overall, the frequency of codons ending with A/T or A + T-rich codons was much higher compared to other synonymous codons.

A complete set of 22 *tRNAs* (with 1 encoded for each amino acid and 2 for leucine and serine) were present in *P. clavata* and *B. amblyops* mitogenome. *H. venator* contained an extra *trnQ* in addition to typical 22 tRNA genes. The size of tRNA ranged from 56 bp to 76 bp (Tables S2–S4). Consistent with previously described features in other ant species [17,24,48,51], only *trnS1* lacked the dihydrouridine arm (D-arm), and other tRNAs including a duplicate of *trnQ* in *H. venator* had the typical cloverleaf secondary structure with a dihydrouridine (DHU) arm, anticodon arm, TΨC arm, and aminoacyl stem (Figures S2 and S3).

### 3.2. Non-Coding Regions

The control region (CR), also known as the D-Loop or AT-rich region, is the longest non-coding region in the mitogenome. It has been suggested to be involved in transcription and replication processes [52,53]. The size and location of the control region in ant mitogenomes are highly variable (Tables S2–S4). Among the three new mitogenomes, the largest control region was found in the *P. clavata* mitogenome, measuring 1754 bp in length. This larger control region contributes to the overall size increase of the mitogenome.

**Figure 3:**
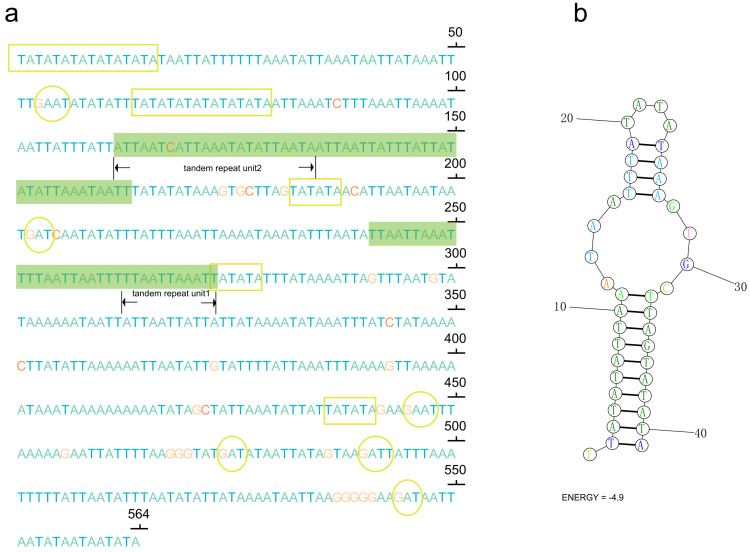
(**a**) Sequences of control region (CR) with microsatellite-like elements (TA)n, G(A)nT motifs, and tandem repeats highlighting in yellow rectangles, yellow cycles, and green shaded boxes, respectively. The letters A, T, C, and G were indicated with different colors (green, blue, red, and orange); (**b**) the inferred stem-loop structures of the tandem repeat motif in the CR of the *P. clavata* mitogenome.

In the control region of *P. clavata*, a notable feature is a large duplicated region consisting of 2 identical copies of 752 bp separated by 17 non-coding nucleotides. Additionally, we observed short tandem duplicated regions with copies of 11 bp and 23 bp units (Figure 3). These tandem repeat motifs have the potential to form stem-and-loop secondary structures when the sequence is folded, which is consistent with the folding pattern observed in animal mitogenomes [54]. The presence of conserved motifs, such as TA(A)n-like stretches, hairpin loop structures, TATA motifs, and G(A)nT motifs, within the control region of *P. clavata* supports their role as initiation sites for replication and transcription (Figure 3).

As *P. clavata* inhabits tropical rainforests near the equator, we speculate that the unique elements within the control region may play a crucial role in regulating transcription and replication of the mitogenome in the specific environment characterized by high temperatures. However, this is a speculative hypothesis, and further comprehensive studies are required to investigate the significance of these inferences and the potential roles of these structures.

**Figure 4:**
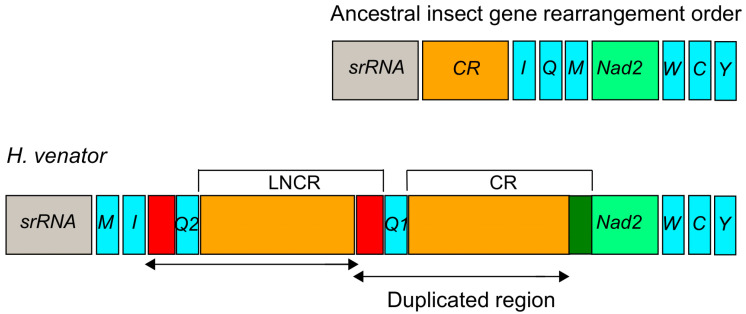
Duplication of the *trnQ* gene and part of *CR* in the region between the srRNA and *ND2* in *H. venator*. The duplicated region is labeled with arrows. *CR* is the abbreviation for the control region and LNCR is the abbreviation for the long non-coding region. The red colors indicated two duplicated regions near to *trnQ* gene.

In *H. venator*, apart from the longest control region (CR) with a length of 500 bp, an additional relatively long non-coding region (LNCR) of 479 bp was found between two *trnQ* genes (Figure 4). Interestingly, a segment of 449 bp within the LNCR was identical to a segment in the CR. This suggests that the LNCR can be identified as a second control region. Furthermore, an 84 bp segment adjacent to *trnQ2* showed sequence identity with the sequences near the 3’ end of the LNCR (Figure 4). These identical sequences suggest that the additional *trnQ* gene was generated through tandem duplication. Consequently, the ancestral insect gene order of *srRNA-CR-I-Q-M-Nad2* [55] underwent rearrangement, resulting in the novel gene order of *srRNA-M-I-Q2-Q1-CR-Nad2* in *H. venator* (Figure 4). The duplicated region in the LNCR is identical, suggesting a recent origin of the duplication event. While tRNA gene duplications have been reported in Hemiptera [27] and Mantodea [29], they have not been previously reported in Formicidae species. Gene duplications often occur near the replication origin, potentially explaining duplication events in that region of *H. venator* [26,27,28,29]. It is anticipated that more novel gene orders will be discovered in the region spanning from the *srRNA* gene to the *Nad2* gene as additional taxa of Formicidae mitogenomes are investigated. Our analysis suggests the presence of a putative copy of the control region in *H. venator*. Multiple control regions are relatively rare in insect mitogenomes but have been reported in parasitic wasps [56]. Overall, the mitogenomes of Poneroid ants exhibit high variation in the control region, consistent with previous studies that have highlighted it as a hotspot for gene rearrangements [27,28].

### 3.3. Gene Rearrangements

**Figure 5:**
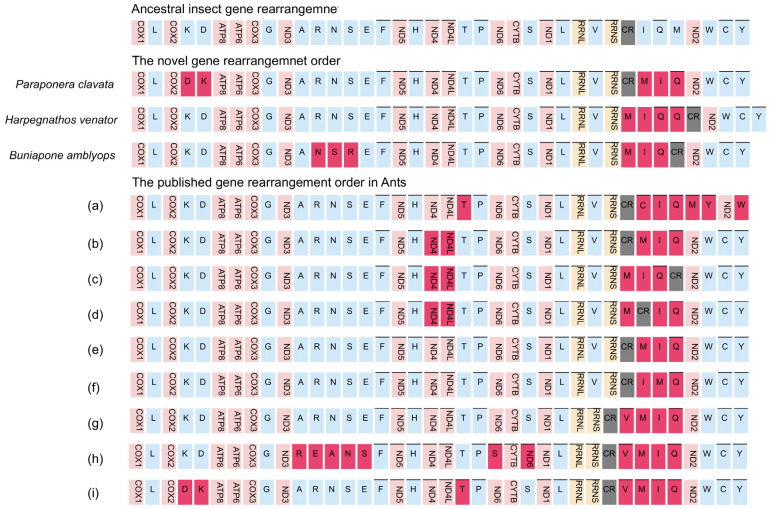
Types of gene rearrangement in Formicidae. The transcribed direction is from left to right except for those overlined, which have the opposite transcriptional overline. PCGs and rRNA genes are shown by standard abbreviations, while tRNA genes are denoted by single letters. PCGs are indicated in red, rRNA in yellow, tRNA in blue, and control region in grey. The ancestral insect gene order is shown on the top, then are the novel gene rearrangements for three newly sequenced mitogenomes, and the published gene rearrangements orders. (**a**) Amblyoponinae (*Stigmatomma silvestrii*); (**b**) Proceratiinae (*Proceratium itoi*); (**c**) Ponerinae (*Ectomomyrmex javanus*); (**d**) Ponerinae (*Cryptopone sauteri*); (**e**) Pseudomyrmecinae (*Tetraponera aethiops*, *Pseudomyrmex gracilis*), Dolichoderinae (*Ochetellus glaber, Linepithema humile, Dolichoderus sibiricus*), Formicinae (*Lasius spathepus, Formica sinae*); (**f**) Formicinae (*Camponotus japonicus*); (**g**) Myrmicinae (*Carebara diversa*); (**h**) Myrmicinae (*Monomorium pharaonis*); (**i**) Myrmicinae (*Pristomyrmex punctatus*).

The occurrence of gene rearrangements is common in Hymenoptera mitogenomes [25,55,57,58]. While most mitochondrial genes in Formicidae follow a conserved pattern similar to the putative ancestral insect mitogenome, different types of gene rearrangements have been documented in various Formicidae subfamilies. For instance, Myrmicinae [59], Formicinae [60], and Dolichoderinae [48] have been reported to exhibit gene rearrangements. Taking into account previously published Formicidae mitogenomes, we identified a total of 12 gene rearrangement types (Figure 5).

The gene cluster *trnM-trnI-trnQ*, located adjacent to the control region, has been identified as a hotspot for gene rearrangements in insect mitogenomes [26,28,58]. Within this region, three different types of gene clusters have been observed. The most frequent type, accounting for 83% of cases (10 out of 12 types), is the *trnM-trnI-trnQ* order, which is likely an ancestral order in Formicidae. The other two types are the *trnI-trnQ-trnM* order found in the Amblyoponinae subfamily and the *trnI-trnM-trnQ* order found in the Formicinae subfamily (Figure 5). The sequencing of our new mitogenomes consistently revealed a gene order of *trnM-trnI-trnQ*, which corresponds to the putative ancestral gene order in Formicidae [48]. This finding suggests that the gene rearrangement of *trnM-trnI-trnQ* in the common ancestor of ants differs from the ancestral gene rearrangements found in other insects (*trnI-trnQ-trnM*) [23].

The most remarkable gene rearrangement observed in *H. venator* is the duplication of the *CR-trnQ*, and a putative copy of the control region. The gene order in *H. venator* is *trnM-trnI-trnQ2-trnQ1-CR*. The identical sequences of the two *trnQ* genes suggest that this duplication event is relatively recent. To our knowledge, this is the first report of tRNA duplication in a Formicidae species.

Previous studies have indicated that most gene duplications tend to occur within the control region, potentially resulting from slipped-strand mispairing, which is consistent with our findings [28,29,57,58]. In *P. clavata*, we observed a translocation change from *trnK-trnD* to *trnD-trnK*. This is a novel rearrangement among the Poneroid clade species. Although inversions between *trnK* and *trnD* have been reported in other insects [17], they have only been observed in one ant species (*Pristomyrmex punctatus*) [61], suggesting a possible case of convergent evolution (Figure 5).

We also identified a novel rearrangement of gene blocks (*trnA-trnN-trnS-trnR-trnE-trnF*) in *B. amblyops*, which differs from the ancestral order of *trnA-trnR-trnN-trnS-trnE-trnF* observed in most insects (Figure 5).

In summary, the mitochondrial gene rearrangements identified in *P. clavata, H. venator*, and *B. amblyops* have been reported for the first time and are novel findings in Formicidae mitogenomes. Of particular interest is the duplication of the *trnQ* gene observed in the species. These findings highlight the potential presence of abundant gene rearrangements in Poneroid ants, which may have been overlooked due to the limited availability of complete mitochondrial genomes. The identification of more mitogenomes from Poneroid ants is necessary to fully understand the patterns of gene rearrangements in this group.

**Figure 6:**
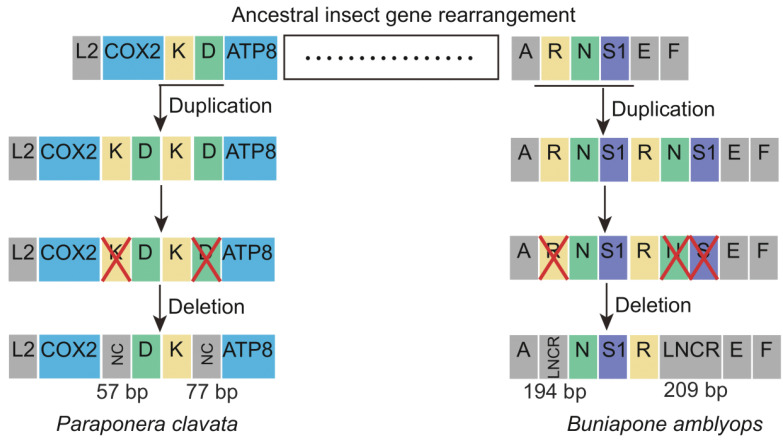
The hypothetical process of gene rearrangements in the mitogenome of *Paraponera clavata* and *Buniapone amblyops*. The size of the gene is not scaled. The horizontal line indicates duplications of gene blocks. The cross mark indicates the partially random loss of the duplicated genes. Different types of genes are labeled with different colors. NC: non-coding region; LNCR: long non-coding region.

The novel gene rearrangements observed in *P. clavata* and *B. amblyops* mitogenomes can be explained by the slipped-strand mispairing model and the tandem duplication/random loss (TDRL) model [32]. According to the TDRL model, the gene rearrangement process in *P. clavata* can be inferred as follows (Figure 6). Initially, a tandem gene duplication event occurred, resulting in a repeating gene cluster of *trnK-trnD-trnK-trnD*. Subsequently, random loss of the first repeat of *trnK* and the second repeat of *trnD* occurred, creating a 57 bp and 77 bp non-coding region and generating a new gene order of *trnD-trnK*. The gene rearrangements in *B. amblyops* followed a similar pattern. Tandem duplication of the gene cluster *trnR-trnN-trnS1* resulted in a repeating gene order of *trnR-trnN-trnS1-trnR-trnN-trnS1*. This was followed by the loss of *trnR* in the upstream repeat and the loss of *trnN* and *trnS1* in the downstream repeat, leading to a novel gene order in this region. Two long non-coding regions (LNCRs) of 194 bp and 209 bp were identified in this region, further supporting the proposed novel gene rearrangement based on the TDRL model.

### 3.4. Heterogeneous Sequence Divergence and Phylogenetic Analysis

The rich diversity of ant species is distributed across 17 subfamilies, which are grouped into 3 major clades: Formicoid, Leptanilloid, and Poneroid [12]. Within the Poneroid clade, there are six subfamilies: Agroecomyrmecinae, Apomyrminae, Amblyoponinae, Ponerinae, Proceratiinae, and Paraponerinae [12]. The exact relationship between Paraponerinae and other subfamilies within the Poneroid clade is still uncertain. Previous analyses of 28 S rRNA gene sequences supported a sister relationship between Paraponerinae and Proceratiinae, placing them as distinct lineages within the Poneroid clade [3]. Other studies analyzing ultraconserved elements and nuclear genes supported the close relationship of Paraponerinae with (Agroecomyrmecinae + Amblyoponinae) and (Agroecomyrmecinae + Ponerinae), respectively [4,5].

**Figure 7:**
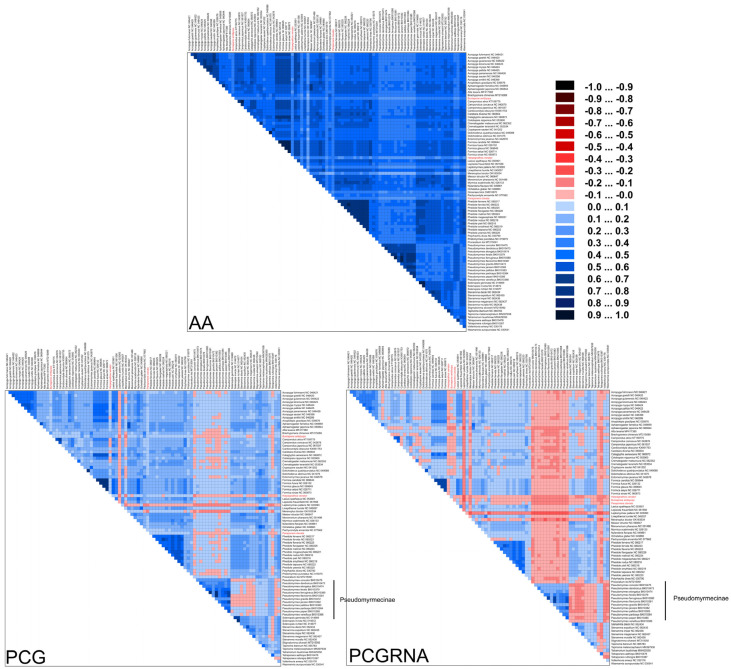
Heterogeneous sequence divergence within Formicidae mitochondrial genomes based on amino acids of protein-coding genes (AA), nucleotides of protein-coding genes (PCG), and nucleotides of protein-coding genes and rRNA genes (PCGRNA). The mean similarity score between sequences is represented by a colored square based on AliGROOVE scores, the scores range from −1 (red) when distances are very different from the average for the entire data matrix to +1 (deep blue) for distances that match the average of the entire matrix. This provides a direct evaluation of sequence heterogeneity for species or clades with respect to the full dataset. The three newly sequenced ant species were highlighted. The species from the subfamily Pseudomyrmecinae demonstrated high sequence heterogeneity.

Ants have a deep evolutionary history of around 150 million years [6], leading to substantial variation in substitution patterns among sites. We hypothesize that accelerated substitution rates and compositional heterogeneity in ant mitochondrial genomes may have a significant impact on phylogenetic inference in Formicidae. To investigate this, we used the AliGROOVE [42] software (v.1.08) to analyze the compositional heterogeneity of protein-coding genes (PCG), PCG and RNA genes (PCGRNA), and amino acid (AA) sequences. The results showed that Formicidae mitogenomes exhibit high levels of sequence heterogeneity, with *Paraponera clavata* and species from the subfamily Pseudomyrmecinae displaying exceptionally high heterogeneity (Figure 7).

To assess the phylogenetic relationships of ants, we performed phylogenetic analyses using maximum likelihood (ML) and Bayesian inference (BI) methods based on PCG, PCGRNA, and AA sequence alignments. The positions of species with higher degrees of sequence heterogeneity were found to be unstable in the reconstructed trees, indicating the presence of potential long-branch attraction (LBA) artifacts.

**Figure 8:**
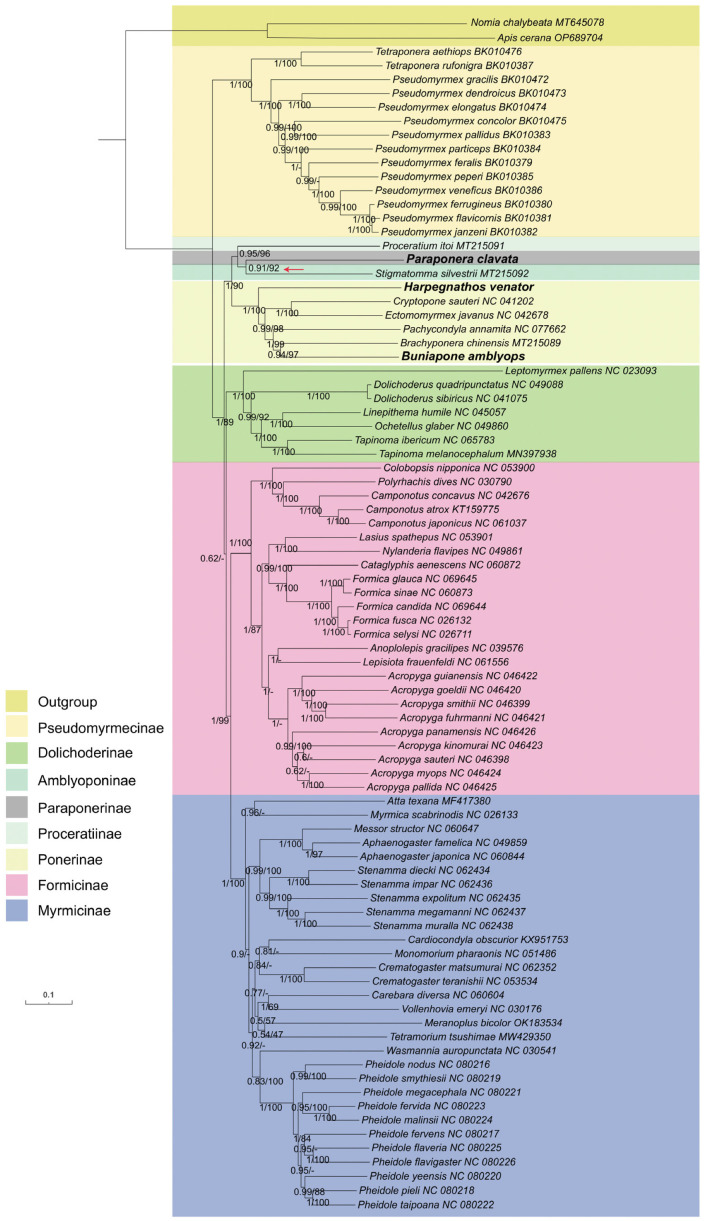
Phylogenetic trees inferred from Bayesian inference and maximum likelihood analyses based on the PCGRNA dataset. The GenBank accession numbers of all species are shown after species names. The numbers in the nodes are Bayesian posterior probabilities (BPP) and bootstrap values (BSV). “-” indicates the node is not supported by the ML tree. The node of Paraponerinae and Amblyoponinae is indicated by a red arrow.

Comparisons between the BI trees constructed with PhyloBayes [46] and the ML trees constructed with IQ-tree 2 [44] revealed that the BI and ML trees obtained from the PCGRNA dataset were mostly congruent and supported with high nodal support (Figure 8). Both methods supported a close relationship between the subfamilies Paraponerinae and Amblyoponinae (bootstrap value BP = 92, Bayes posterior probabilities PP = 0.91). However, the relationships between Paraponerinae and other subfamilies appeared to be unstable in the topology results obtained from other datasets, displaying low support values in both posterior probabilities (PPs) and bootstrap percentages (BSs).

Based on these findings, we conclude that the incorporation of the rRNA gene could be beneficial in phylogenetic analysis within ant mitogenomes, as it may provide additional information for phylogenetic inference. Therefore, we consider the tree obtained from Bayesian inference based on the PCGRNA dataset as our best estimation (Figure 8). The newly sequenced species *H. venator* and *B. amblyops* were placed within the subfamily Ponerinae, and their locations were consistent in both ML and BI trees. The subfamily Ponerinae was found to be monophyletic, with the following relationships between the seven genera: (((Buniapone+ Brachyponera) + Pachycondyla) + (Cryptopone + Ectomomyrmex)) + Harpegnathos. These relationships were strongly supported (BS > 97, PP > 0.94). Furthermore, a close relationship between the subfamilies Myrmicinae and Formicinae was strongly supported (BS = 99, PP = 1)

In our study, the phylogenetic analysis using ML and BI trees with PCGRNA data showed a close relationship between the subfamilies Paraponerinae and Amblyoponinae, consistent with previous studies using nuclear data [4]. However, caution should be exercised in drawing conclusions as long-branch attraction was found in the subfamily Paraponerinae due to deep nodes and high sequence heterogeneity of *P. clavata*. The position of *P. clavata* was found to be unstable in trees obtained from different datasets (Figures S4 and S5). Since *P. clavata* is the sole existing species of Paraponerinae and obtaining mitochondrial genomes from more taxa of Paraponerinae is not possible, we anticipate that high-quality whole genome sequencing data will help resolve the controversy surrounding Paraponerinae in the future [62].

The monophyly of subfamilies is well supported using ML and BI methods based on three datasets (PCG, PCGRNA, and AA), and the monophyly of the Poneroid clade is universally accepted, as indicated by previous studies [3,4,5,8,9,63]. However, the monophyly of the Formicoid clade, supported by nuclear DNA or morphological data [5,12,63,64,65,66], was not recovered. The mitogenomes of ants exhibit significantly high sequence heterogeneity, which limits the resolving power of phylogenetic inference using mitochondrial data alone. We suspect that the presence of shared sequence compositional biases led to an increase in homoplasy, thereby generating a non-phylogenetic signal. Furthermore, lineage-specific evolutionary rates in molecular divergence could have a significant impact on phylogenetic reconstruction. Future data obtained from whole genome alignments will provide robust phylogenetic information to resolve the higher-level phylogeny within Formicidae [67,68].

## 4. Conclusions

In this study, we successfully sequenced and obtained the complete mitogenomes of the only extant species *Paraponera clavata* from the subfamily Paraponerinae, as well as two species from the subfamily Ponerinae (*Harpegnathos venator* and *Buniapone amblyops*). These mitogenomes revealed novel gene rearrangements, including *srRNA-M-I-Q2-Q1-CR-Nad2* in *H. venator*, *COX1-trnD-trnK-ATP8* in *P. clavata*, and *trnA-trnN-trnS-trnR-trnE-trnF* in *B. amblyops*. The duplication of tRNA genes was reported for the first time in Formicidae. A plausible explanation for these gene rearrangements can be given by the slipped-strand mispairing model and tandem duplication/random loss (TDRL) model. The control region (CR) of the *P. clavata* mitogenome exhibited a large duplicated region including tandem repeats and conserved motifs. Phylogenetic analysis using both BI and ML methods supported the monophyly of the Poneroid clade and suggested a sister group relationship between the subfamilies Paraponerinae and Amblyoponinae. However, long-branch attraction was observed in Paraponerinae, leading to uncertainty in their phylogenetic positions. Additional data are required to further elucidate the relationships between Paraponerinae and other subfamilies within Formicidae.

## Figures and Tables

**Table 1:** Base composition of three new mitogenomes.

Species	A + T (%)				AT Skew				GC Skew			
	MT	PCGs	rRNAs	CR	MT	PCGs	rRNAs	CR	MT	PCGs	rRNAs	CR
*H. venator*	81.4	78.6	85.2	93.6	0.044	−0.141	0.093	−0.013	−0.342	0.083	−0.453	−0.342
*B. amblyops*	80.1	78.2	84.2	87.2	0.087	−0.154	0.113	0.089	−0.43	0.062	−0.511	−0.43
*P. clavata*	82.1	79	85.7	91.8	−0.007	−0.134	0.012	0.025	−0.321	0.018	−0.4	−0.321

## Data Availability

The data that support the findings of this study have been deposited into the CNGB Sequence Archive (CNSA) of China National GeneBank DataBase (CNGBdb) with accession number CNP0004849. The GenBank accession number associated with this study is OR395164, OR633237 and OR633238.

## Supplementary Materials

The following supporting information can be downloaded at https://www.mdpi.com/article/10.3390/life13102068/s1, Figure S1: The sequences alignment of *trnP* gene of *B. amblyops* and other ant species. The arrow indicates the edge of the assembled mitochondrial sequences of *B. amblyops*, which is located in the 50 bp position. The failure to identify *trnP* using Mitos and Mitoz is caused by the broken at the middle of sequences of *trnP* gene; Figure S2: Inferred secondary structure of 22 tRNAs of the *P. clavata* mitochondrial genome; Figure S3: Inferred secondary structure of trnS1 of the H. venator and *B. amblyops* mitochondrial genomes. Both lacked the dihydrouridine arm (D-arm); Figure S4: Phylogenetic trees inferred from maximum likelihood analyses of PCG dataset. *Paraponera clavata* is close to *Proceratium itoi* from the subfamily Proceratiinae with low nodal support (BS = 43); Figure S5: Phylogenetic trees inferred from maximum likelihood analyses of AA dataset. *Paraponera clavata* is close to subfamily Ponerinae with weak nodal support (BS = 79); Table S1: Taxonomic information, size, and GenBank accession numbers of mitochondrial genomes from Poneroid and part of Formicoid ants; Table S2: Characteristics of the *Paraponera clavata* mitochondrial genome; Table S3: Characteristics of the *Harpegnathos venator* mitochondrial genome. Table S4. Characteristics of the *Buniapone amblyops* mitochondrial genome.
