# Supplementary material for: Novel tRNA Gene Rearrangements in the Mitochondrial Genomes of Poneroid Ants and Phylogenetic Implication of Paraponerinae (Hymenoptera: Formicidae)
Source: Life (Basel). 2023 Oct 16;13(10):2068. doi: 10.3390/life13102068 (PMC10608118; doi:10.3390/life13102068)
Supplement: Supplementary file 1 [file life-13-02068-s001.zip › life-2620058-supplementary.pdf]

Table S1. Taxonomic information, size, and GenBank accession numbers of mitochondrial genomes from Poneroid and part of Formicoid ants.

**Subfamily of Formicoid clade**	**Species**	**Size (bp)**	**Accession number**	**Reference**
Dolichoderinae	*Tapinoma melanocephalum*	15,499	MN397938	[1]
	Dolichoderus sibiricus	16,086	NC_041075	[2]
	Linepithema humile	15,929	NC_045057	[3]
	Dolichoderus quadripunctatus	16,017	NC_049088	Unpublished
	Ochetellus glaber	16,259	NC_049860	[4]
Formicinae	*Lasius spathepus*	18,951	NC_053901	[5]
	Formica sinae	17,432	NC_060873	[6]
	Camponotus japonicus	16,422	NC_061037	[7]
Myrmicinae	*Monomorium pharaonis*	15,942	NC_051486	[8]
	Solenopsis invicta	15,549	NC_014672	[9]
	Carebara diversa	15,487	NC_060604	Unpublished
	Messor structor	17,628	NC_060647	[10]
	Pristomyrmex punctatus	16,180	NC_015075	[11]
Pseudomyrmecinae	*Pseudomyrmex gracilis*	15,704	BK010472	[12]
	Tetraponera aethiops	15,988	BK010476	[12]
**Subfamily of Poneroid clade**	**Species**	**Size (bp)**	**Accession number**	**Reference**
Amblyoponinae	*Stigmatomma silvestrii*	18,861	MT215092	Unpublished
Proceratiinae	*Proceratium itoi*	15,460	MT215091	Unpublished
Ponerinae	*Pachycondyla annamita*	15,466	NC_077662	[13]
	Ectomomyrmex javanus	15,512	NC_042678	[14]
	Cryptopone sauteri	15,367	NC_041202	[15]
	Brachyponera chinensis	15,789	MT215089	Unpublished
	Harpegnathos venator	**16,089**	OR633237	**This study**
	Buniapone amblyops	**16,621**	OR633238	
**Paraponerinae**	***Paraponera clavata***	**17,018**	OR395164	

Table S2. Characteristics of the *Paraponera clavate* mitochondrial genome.

tRNA	Gene	Start	End	Length (bp)	Strand	Anticodon	Start codon	Stop codon	Intergenic region
	rrnS	11	849	839	+				20
Val	*trnV*	869	932	64	+	TAC			16
	rrnL	948	2,300	1,353	+				-23
Leu	*trnL*	2,278	2,342	676	+	TAG			0
	ND1	2,343	3,317	975	+		ATA	TAA	-21
Ser	*trnS*	3,297	3,367	71	-	TGA			25
	CYTB	3,393	4,514	1,122	-		ATG	TAA	104
	ND6	4,619	5,164	546	-		ATG	TAA	10
Pro	*trnP*	5,175	5,245	71	+	TGG			3
Thr	*trnT*	5,249	5,318	70	-	TGT			81
	ND4L	5,400	5,687	288	+		ATT	TAG	-7
	ND4	5,681	7,018	1,338	+		ATT	TAA	2
His	*trnH*	7,021	7,085	67	+	TGT			-3
	ND5	7,083	8,747	1,665	+		ATT	TAG	1
Phe	*trnF*	8,749	8,814	66	+	GAA			-2
Glu	*trnE*	8,813	8,886	74	-	TTC			6
Ser	*trnS*	8,892	8,951	60	-	TCT			-4
Asn	*trnN*	8,948	9,020	73	-	GTT			4
Arg	*trnR*	9,027	9,094	68	-	TCG			5
Ala	*trnA*	9,102	9,167	66	-	TGC			46
	ND3	9,214	9,552	339	-		ATA	TAA	0
Gly	*trnG*	9,553	9,621	69	-	TCC			8
	COX3	9,630	10,418	789	-		ATG	TAA	-1
	ATP6	10,418	11,079	662	-		ATA	TA	-4
	ATP8	11,076	11,234	159	-		ATA	TAA	77
Lys	*trnK*	11,312	11,379	68	-	TTT			-2
Asp	*trnD*	11,378	11,444	67	-	GTC			57
	COX2	11,502	12,179	690	-		ATC	TAA	0
Leu	*trnL*	12,180	12,244	65	-	TAA			24
	COX1	12,269	13,798	1,530	-		ATG	TAA	7
Tyr	*trnY*	13,806	13,871	66	+	GTA			6
Cys	*trnC*	13,880	13,946	677	+	GCA			17
Trp	*trnW*	13,966	14,034	69	-	TCA			22
	ND2	14,059	15,075	1,017	-		ATT	TAA	-28
Gln	*trnQ*	15,048	15,116	69	+	TTG			4
Ile	*trnI*	15,121	15,190	70	-	GAT			4
Met	*trnM*	15,195	15,264	70	-	CAT			

Table S3. Characteristics of the *Harpegnathos venator* mitochondrial genome.

tRNA	Gene	Start	End	Length (bp)	Strand	Anticodon	Start codon	Stop codon	Intergenic region
Ile	*trnI*	18	86	69	-	GAT			11
Met	*trnM*	98	165	71	-	CAT			-28
	rrnS	138	967	830	+				10
Val	*trnV*	978	1044	67	+	TAC			14
	rrnL	1059	2,398	1340	+				-25
Leu	*trnL*	2,374	2,443	70	+	TAG			3
	ND1	2,447	3,412	966	+		ATT	TAA	-18
Ser	*trnS*	3,395	3,466	72	-	TGA			58
	CYTB	3,525	4,628	1104	-		ATT	TAA	36
	ND6	4,665	5,219	555	-		ATG	TAA	4
Pro	*trnP*	5,224	5,289	66	+	TGG			16
Thr	*trnT*	5,306	5,379	74	-	TGT			15
	ND4L	5,395	5,682	288	+		ATA	TAA	44
	ND4	5,727	7,046	1320	+		ATG		2
His	*trnH*	7,049	7,114	66	+	TGT			6
	ND5	7,121	8,750	1630	+		ATT	T	27
Phe	*trnF*	8,778	8,843	66	+	GAA			15
Glu	*trnE*	8,859	8,914	56	-	TTC			9
Ser	*trnS*	8,924	8,985	62	-	TCT			-2
Asn	*trnN*	8,984	9,053	70	-	GTT			44
Arg	*trnR*	9,098	9,159	62	-	TCG			5
Ala	*trnA*	9,165	9,231	67	-	TGC			1
	ND3	9,233	9,568	336	-		ATC	TAA	9
Gly	*trnG*	9,578	9,645	68	-	TCC			38
	COX3	9,684	10,472	789	-		ATG	TAA	3
	ATP6	10,476	11,141	666	-		ATG	TAA	-7
	ATP8	11,135	11,293	159	-		ATC	TAA	0
Asp	*trnD*	11,294	11,363	70	-	GTC			0
Lys	*trnK*	11,364	11,439	76	-	TTT			10
	COX2	11,450	12,130	681	-		ATT	TAA	0
Leu	*trnL*	12,131	12,202	72	-	TAA			-5
	COX1	12,198	13,730	1533	-		ATG	TAA	0
Tyr	*trnY*	13,731	13,796	66	+	GTA			8
Cys	*trnC*	13,805	13,872	68	+	GCA			-8
Trp	*trnW*	13,865	13,931	67	-	TCA			9
	ND2	13,941	14,889	949	-		ATT	T	500
Gln	*trnQ*	15,390	15,458	69	+	TTG			479
Gln	*trnQ*	15,938	16,006	69	+	TTG			

Table S4. Characteristics of the *Buniapone amblyops* mitochondrial genome.

tRNA	Gene	Start	End	Length (bp)	Strand	Anticodon	Start codon	Stop codon	Intergenic region
Thr	*trnT*	49	120	72	-	TGT			4
	ND4L	125	421	297	+		ATA	TAA	8
	ND4	430	1,791	1,362	+		ATG	TAA	13
His	*trnH*	1,805	1,873	69	+	TGT			-3
	ND5	1,871	3,568	1,698	+		ATT	TAA	8
Phe	*trnF*	3,577	3,645	69	+	GAA			11
Glu	*trnE*	3,657	3,721	65	-	TTC			209
Arg	*trnR*	3,931	3,994	64	-	TCG			68
Ser	*trnS*	4,063	4,121	59	-	TCT			84
Asn	*trnN*	4,206	4,280	75	-	GTT			194
Ala	*trnA*	4,475	4,538	64	-	TGC			38
	ND3	4,577	4,927	351	-		ATT	TAA	96
Gly	*trnG*	5,024	5,095	72	-	TCC			67
	COX3	5,163	5,945	783	-		ATG	TAA	-8
	ATP6	5,938	6,612	675	-		ATA	TAA	43
	ATP8	6,656	6,823	168	-		ATT	TAA	0
Asp	*trnD*	6,824	6,896	73	-	GTC			-2
Lys	*trnK*	6,895	6,966	72	-	TTT			44
	COX2	7,011	7,694	684	-		ATT	TAA	0
Leu	*trnL*	7,695	7,765	71	-	TAA			0
	COX1	7,766	9,294	1,529	-		ATG	TA	11
Tyr	*trnY*	9,306	9,371	66	+	GTA			8
Cys	*trnC*	9,380	9,447	68	+	GCA			-8
Trp	*trnW*	9,440	9,508	69	-	TCA			93
	ND2	9,602	10,481	880	-		ATC	T	554
Gln	*trnQ*	11,036	11,109	74	+	TTG			13
Ile	*trnI*	11,123	11,190	68	-	GAT			12
Met	*trnM*	11,203	11,273	71	-	CAT			-30
	rrnS	11244	12094	851	+				1
Val	*trnV*	12096	12164	69	+	TAC			31
	rrnL	12196	13,600	1,405	+				-25
Leu	*trnL*	13,576	13,641	66	+	TAG			18
	ND1	13,660	14,595	936	+		ATA	TAA	17
Ser	*trnS*	14,613	14,681	69	-	TGA			172
	CYTB	14,854	15,957	1,104	-		ATT	TAA	49
	ND6	16,007	16,534	528	-		ATG	TAA	31
Pro	*trnP*	16,566	16,633	68	+	TGG			

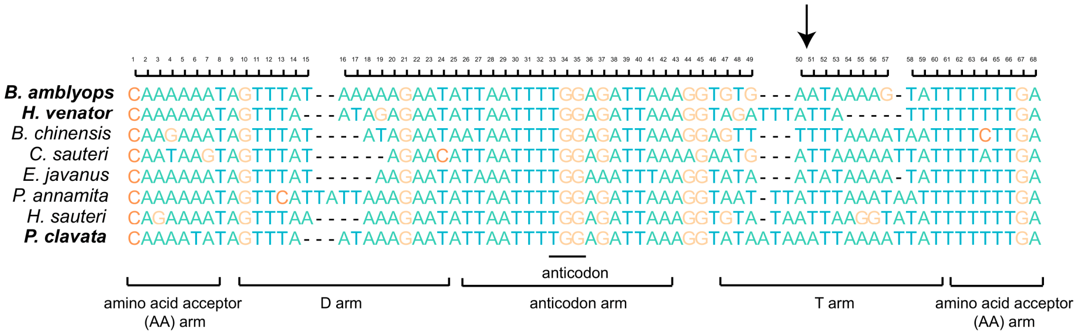

Figure S1. The sequences alignment of *trnP* gene of *B. amblyops* and other ant species. The arrow indicates the edge of the assembled mitochondrial sequences of *B. amblyops*, which located in the 50 bp position. The failure to identify *trnP* using Mitos and Mitoz is caused by the broken at the middle of sequences of *trnP* gene.

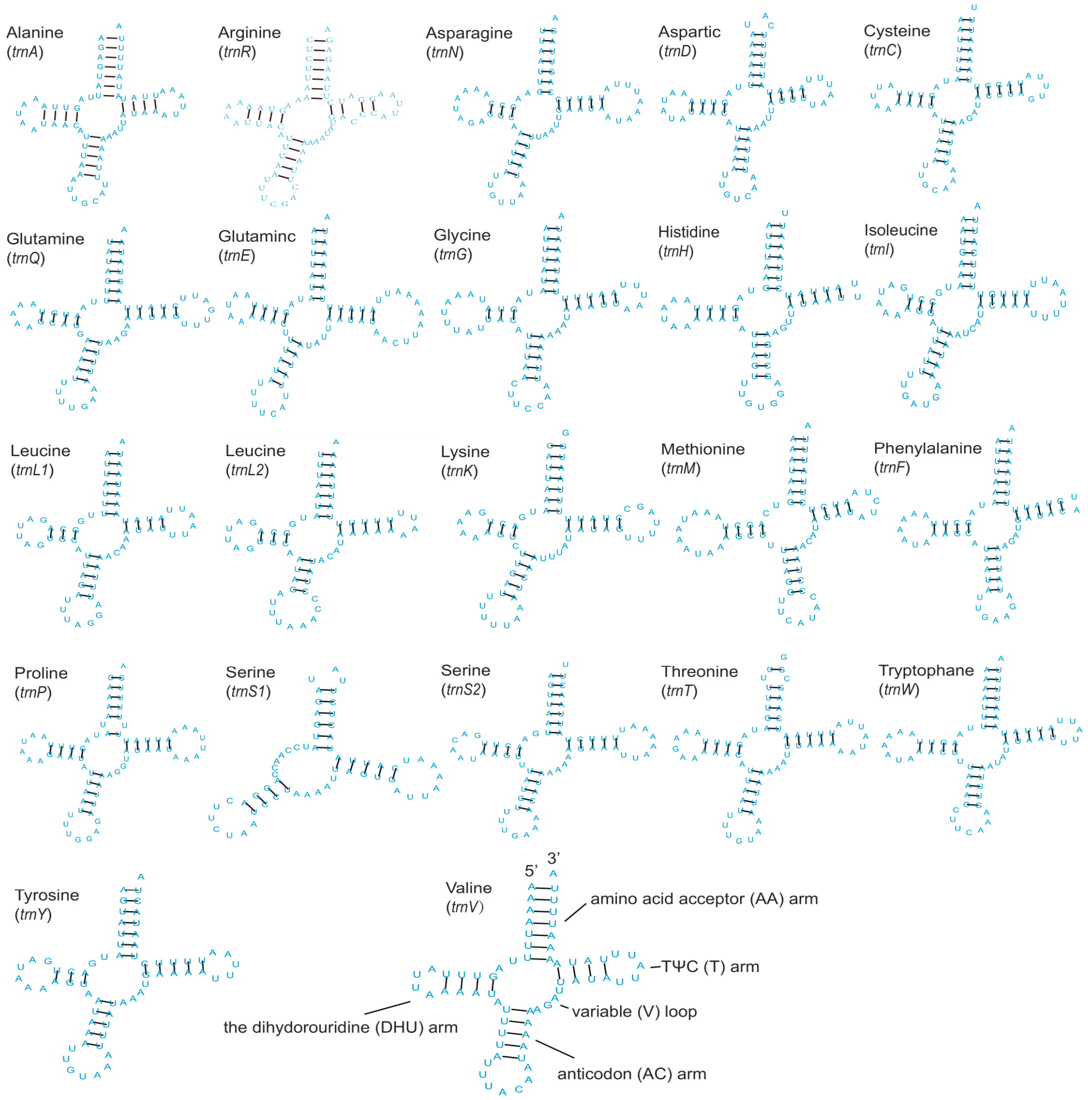

Figure S2. Inferred secondary structure of 22 tRNAs of the *P. clavate* mitochondrial genome.

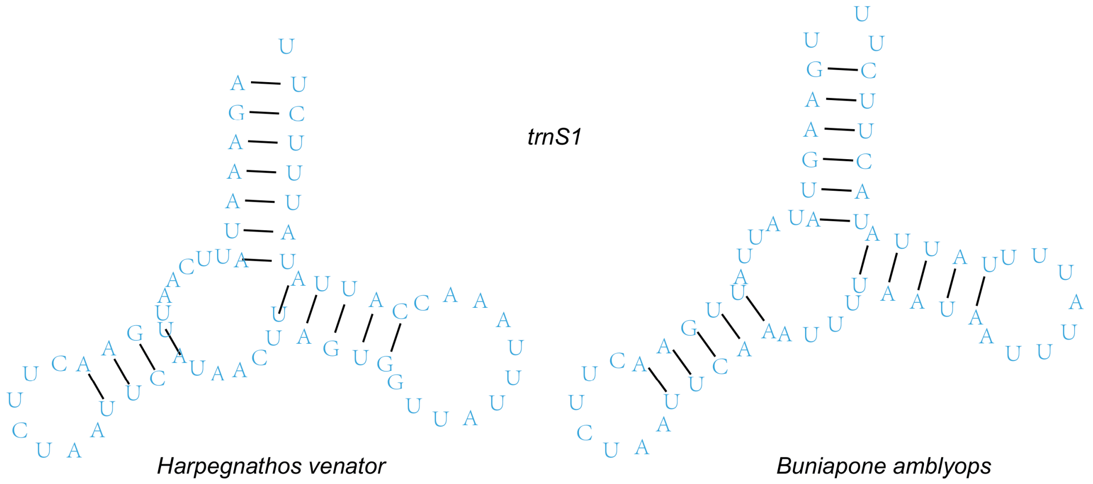

Figure S3. Inferred secondary structure of *trnS1* of the *H. venator* and *B. amblyop* mitochondrial genomes. Both lacked the dihydrouridine arm (D-arm).

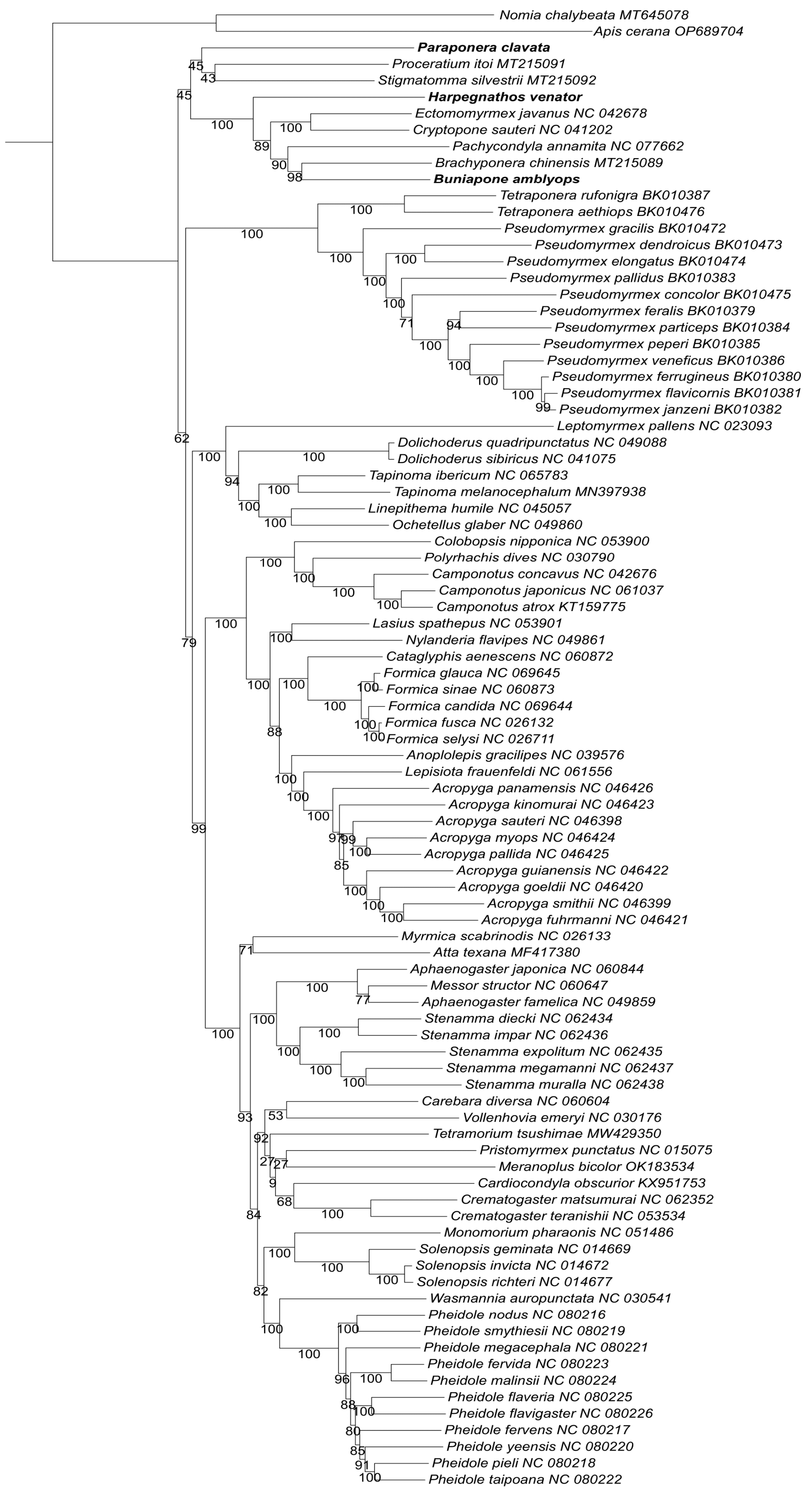

Figure S4. Phylogenetic trees inferred from maximum likelihood analyses of PCG dataset. *Paraponera clavata* is close to *Proceratium itoi* from subfamily Proceratiinae with low nodal support (BS=43)

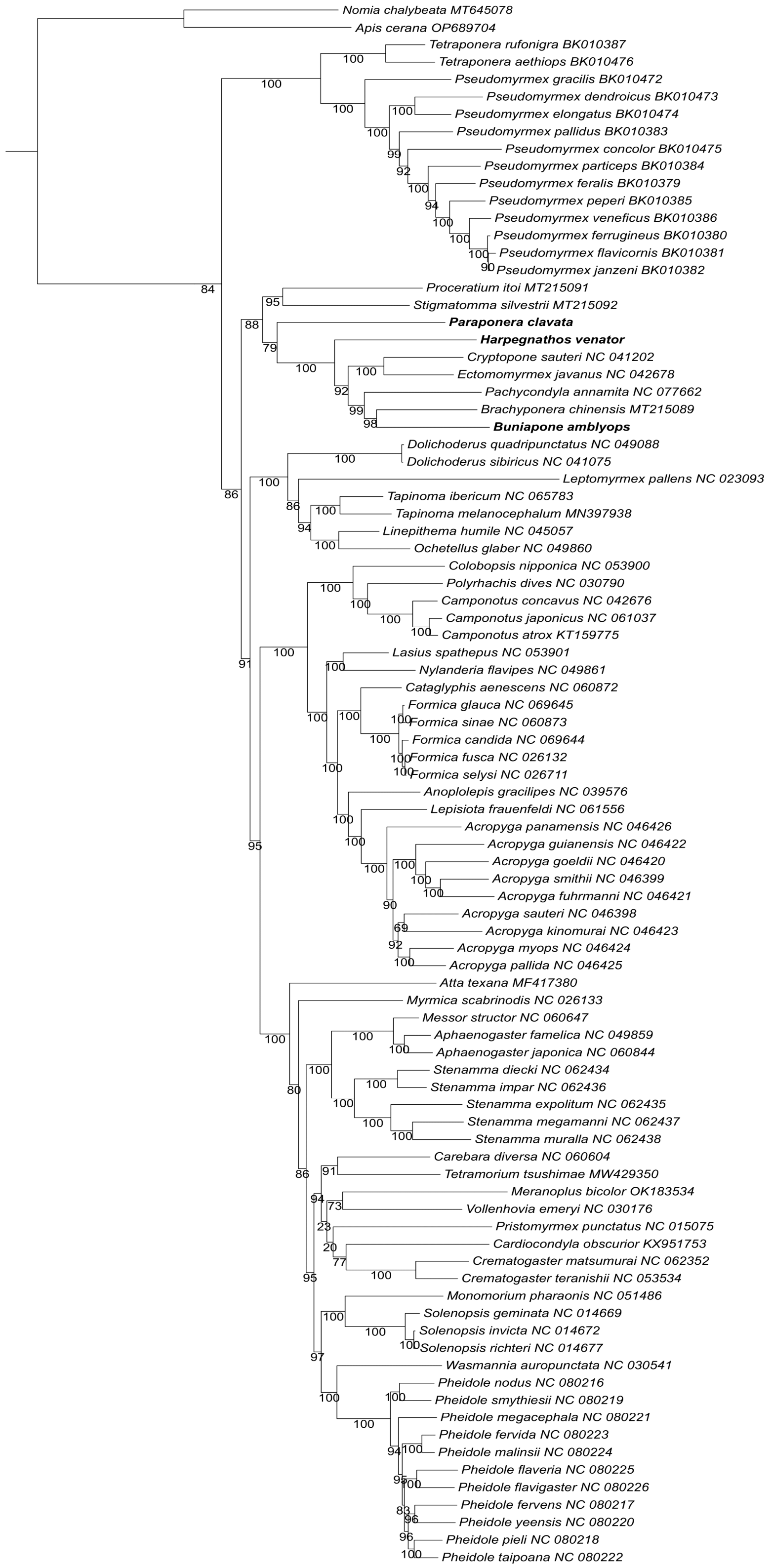

Figure S5. Phylogenetic trees inferred from maximum likelihood analyses of AA dataset. *Paraponera clavata* is close to subfamily Ponerinae with weak nodal support (BS=79).

Reference:

[1] Du Y, Song X, Yu H, Lu Z. Complete mitochondrial genome sequence of Tapinoma melanocephalum (Hymenoptera: Formicidae). Mitochondrial DNA B Resour. 2019 Oct 9;4(2):3448-3449.

[2]Jonghyun Park, Woochan Kwon & Jongsun Park (2019) The complete mitochondrial genome of Siberian odorous ant, Dolichoderus sibiricus Emery, 1889 (Hymenoptera: Formicidae), Mitochondrial DNA Part B, 4:1, 525-526.

[3] Zhao E, Bi G, Yang J, Zhang Z, Liu G, Du Q, Shang E. Complete mitochondrial genome of the argentine ant, Linepithema humile (Hymenoptera: Formicidae). Mitochondrial DNA A DNA Mapp Seq Anal. 2017 Mar;28(2):210-211.

[4] Park J, Xi H, Park J. The complete mitochondrial genome of Ochetellus glaber (Mayr, 1862) (Hymenoptera:Formicidae). Mitochondrial DNA B Resour. 2019 Dec 11;5(1):147-149.

[5] Park J, Park J. Complete mitochondrial genome of the jet ant Lasius spathepus Wheeler, W.M., 1910 (Formicidae; Hymenoptera). Mitochondrial DNA B Resour. 2021 Feb 15;6(2):505-507.

[6] Zhang XM, Li T, Liu X, Xu ZH. Characterization and Phylogenetic Implication of Complete Mitochondrial Genome of the Medicinal Ant Formica sinae (Hymenoptera: Formicidae): Genomic Comparisons in Formicidae. J Med Entomol. 2022 Nov 16;59(6):1971-1979.

[7] Shen S, Li W. Phylogenetic relationship and characterization of the complete mitochondrial genome of Camponotus japonicus (Hymenoptera: Formicoidea: Formicidae). Mitochondrial DNA B Resour. 2022 Apr 22;7(4):686-688.

[8] Gao Q, Xiong Z, Larsen RS, Zhou L, Zhao J, Ding G, Zhao R, Liu C, Ran H, Zhang G. High-quality chromosome-level genome assembly and full-length transcriptome analysis of the pharaoh ant Monomorium pharaonis. Gigascience. 2020 Dec 15;9(12):giaa143.

[9] Gotzek D, Clarke J, Shoemaker D. Mitochondrial genome evolution in fire ants (Hymenoptera: Formicidae). BMC Evol Biol. 2010 Oct 7;10:300.

[10] Zhang XM, Han X, Liu X, Xu ZH. Characterization of the complete mitochondrial genome of a harvesting ant Messor structor (Hymenoptera: Formicidae: Myrmicinae). Mitochondrial DNA B Resour. 2022 Jun 2;7(6):933-935.

[11] Hasegawa, E.; Kobayashi, K.; Yagi, N.; Tsuji, K. Complete mitochondrial genomes of normal and cheater morphs in the parthenogenetic ant Pristomyrmex punctatus (Hymenoptera: Formicidae). Myrmecological News 2011, 15, 85-90.

[12] Vieira GA, Prosdocimi F. Accessible molecular phylogenomics at no cost: obtaining 14 new mitogenomes for the ant subfamily Pseudomyrmecinae from public data. PeerJ. 2019 Jan 24;7:e6271.

[13] Lin X, Song N. The First Complete Mitochondrial Genome of the Genus *Pachycondyla* (Formicidae, Ponerinae) and Insights into the Phylogeny of Ants. Genes (Basel). 2023 Jul 26;14(8):1528.

[14] Jonghyun Park, Woochan Kwon & Jongsun Park (2019) The complete mitochondrial genome of *Ectomomyrmex javanus* Mayr, 1867 (Hymenoptera: Formicidae), Mitochondrial DNA Part B, 4:1, 1636-1637.

[15] Jonghyun Park, Woochan Kwon & Jongsun Park (2019) The complete mitochondrial genome of *Cryptopone sauteri* Wheeler, W.M., 1906 (Hymenoptera: Formicidae), Mitochondrial DNA Part B, 4:1, 614-615.
